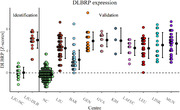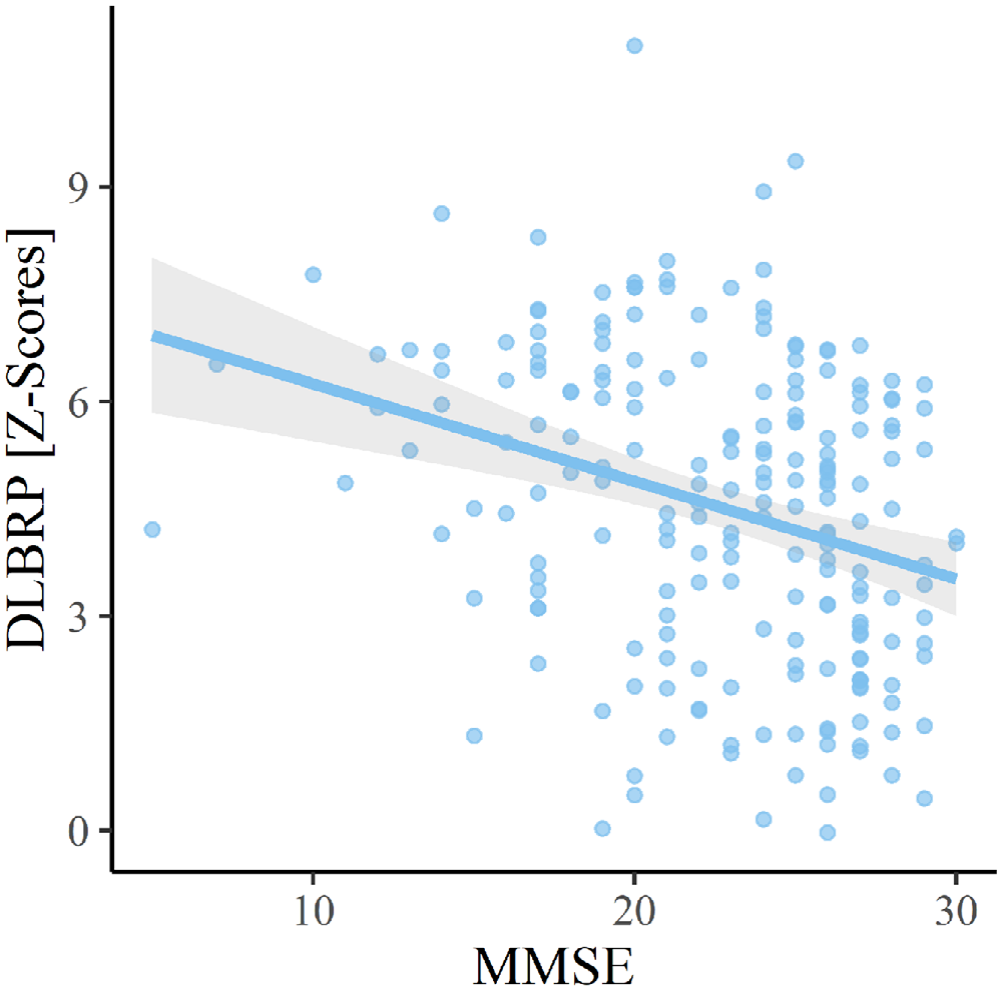# Multicentre validation of the dementia with Lewy bodies‐related metabolic brain pattern

**DOI:** 10.1002/alz.094112

**Published:** 2025-01-09

**Authors:** Matej Perovnik, Urban Simončič, Hana Kos, Jan Jamšek, Milica G Kramberger, Matthias Brendel, Valle Camacho, Rik Vandenberghe, Valentina Garibotto, Miguel A Ochoa‐Figueroa, Alessandro Padovani, Silvia Morbelli, Daniel Ferreira, Maja Trošt

**Affiliations:** ^1^ Department for Neurology, UMC Ljubljana, Ljubljana Slovenia; ^2^ Faculty of Mathematics and Physics, University of Ljubljana, Ljubljana Slovenia; ^3^ Medical Faculty, Unversity of Ljubljana, Ljubljana Slovenia; ^4^ Department for Nuclear Medicine, UMC Ljubljana, Ljubljana Slovenia; ^5^ Department of Nuclear Medicine, University Hospital, LMU Klinikum, Munich, Bavaria Germany; ^6^ Nuclear Medicine Department, Hospital de la Santa Creu i Sant Pau, Barcelona Spain; ^7^ University Hospitals Leuven, Leuven Belgium; ^8^ Faculty of Medicine, University of Geneva, Geneva Switzerland; ^9^ Linköping University, Linköping Sweden; ^10^ Neurology Unit, Department of Clinical and Experimental Sciences, University of Brescia, Brescia Italy; ^11^ University of Genoa, Genoa Italy; ^12^ Karolinska Institutet, Stockholm, Sodermanland Sweden

## Abstract

**Background:**

Dementia with Lewy bodies (DLB) is a an a‐synucleinopathy characterized by dementia and a combination of parkinsonism, visual hallucinations, fluctuating cognition or REM sleep behaviour disorder. Specific biomarkers for DLB are lacking. DLB‐related pattern (DLBRP) is a metabolic network imaging biomarker which expression can be quantified on a single patient basis. DLBRP has been identified in few different cohorts but lacks a multicentric validation.

**Methods:**

FDG PET scans from 259 DLB patients and 176 normal controls (NC) were obtained from eight centres from European DLB Consortium (EDLB, 180 patients), Alzheimer’s disease Neuroimaging Initiative (ADNI, 135 NC) and local dataset from Ljubljana (LJU, 79 patients and 41 NC). FDG PET scans were pre‐processed and we used topographic profile rating to calculate the DLBRP expression (i.e. subject scores). Subject scores were compared between groups using t tests and ANOVA and correlated with clinical parameters using Pearson’s correlation coefficient.

**Results:**

Patients from different centres differed in MMSE (p < 0.001), proportion of patients with parkinsonism (p < 0.001) and visual hallucinations (p < 0.001), but not in age (p = 0.15) or sex distribution (p = 0.13). DLB patients from all centres had significantly higher DLBRP expression than NCs (all p < 0.001). We could accurately distinguish between DLB and NC (AUC = 0.983) based on DLBRP expression. We observed a negative correlation between DLBRP expression and MMSE (r = –0.30, p < 0.001). The expression of DLBRP differed among centres, post hoc analysis showed that only patients from Barcelona had significantly lower DLBRP subject scores in comparison to other groups but they were also less cognitively impaired. Patients with visual hallucinations (p < 0.001) had significantly higher DLBRP expression than those without hallucinations and patients with abnormal Datscan™ (p = 0.02) had higher DLBRP expression than those with normal Datscan™. Other core features, levodopa or antidementives did not affect the DLBRP expression.

**Conclusions:**

We showed that DLBRP can accurately distinguish between DLB and NC from multiple centres and different scanners. The DLBRP expression correlated with measurements of cognitive impairment. This validation study confirms that the DLBRP is a robust metabolic imaging biomarker of DLB.